# A transition of power in opioid substitution treatment: Clinic managers' views on the consequences of a patient choice reform

**DOI:** 10.1177/14550725221075003

**Published:** 2022-02-01

**Authors:** Lisa Andersson

**Affiliations:** 59606 Malmö University, Sweden

**Keywords:** benzodiazepines, competition, diversity, empowerment, opioid substitution treatment, patient choice, treatment providers

## Abstract

**Objectives:** Opioid substitution treatment (OST) is often described as a strict and highly regulated treatment method, in which patients have limited influence over their treatment. In 2014, a reform was introduced by the regional council of Skåne in southern Sweden, which allowed OST patients to choose their treatment provider, thus transferring power from care providers to patients. The aim of this study was to examine what this increase in patient influence has meant for the clinics that provide OST in Skåne, and how these clinics have dealt with the new competitive situation that has arisen following the introduction of the reform. **Methods:** The study is based on two waves of semi-structured interviews with clinic managers at all OST clinics in Skåne. **Results:** The clinic managers described the increase in patient influence as a positive change, which had led to the patients being treated with more respect. The competition among clinics was expressed, among other things, in the form of differing views on the prescription of benzodiazepines, which initially gave rise to dissatisfaction among clinics with a more restrictive approach to such prescriptions. The reform did not lead to any clear diversity between clinics, apart from different approaches to the prescription of benzodiazepines. The incentive for competition-based diversity is, however, limited by the strict national regulatory system and by the reimbursement system, which restricts the ways in which clinics can conduct treatment activities. **Conclusion:** OST-clinic managers were largely positive about the increased patient empowerment and the shift in power balance associated with the patient choice reform. The introduction of the reform did not lead to any clear diversity between treatment providers, apart from differing views on the prescription of benzodiazepines, which by some managers was regarded as unfair competition.

It is well established that opioid substitution treatment (OST) using methadone or buprenorphine constitutes the most effective treatment method for opioid dependence. OST reduces mortality and morbidity among heroin-dependent individuals and leads to an improved social situation and reduced criminality (Amato et al., 2011; Bukten et al., 2012; Degenhardt et al., 2011; Fugelstad et al., 2007; Mattick et al., 2014; Sordo et al., 2017). The method has often been criticised, however, for having strict criteria for inclusion and for being implemented in a repressive manner that subjects the patients to discipline and control (Bartoszko, 2021; Bourgois, 2000; Harris & McElrath, 2012; Lalander, 2012; Petersson, 2013b). This has given rise to questions regarding patient empowerment and the ability for patients to influence their treatment, both in the research and in practitioner debates (Bjerge & Nielsen, 2014; Deering et al., 2011; Frank & Bjerge, 2011; Kolind, 2007; McElrath, 2018).

In Sweden, access to OST has long been under-dimensioned, which has previously resulted in queues and difficulties in gaining access to treatment. Policy and practice in Swedish OST are undergoing a transformation, however, towards a more accessible, harm-reduction-oriented approach (Johnson et al., 2017; Monwell, 2019). This shift has been particularly marked in the county of Skåne in southern Sweden (Andersson & Johnson, 2018). In 2014, the Skåne Regional Council, which is responsible for public sector healthcare provision in the region, decided to introduce a reform giving patients the right to choose their treatment provider for OST.^1^ The implementation of a patient choice system means that competition is introduced into a market, as patients then choose which care provider will be paid to meet their treatment needs. OST may be provided by all treatment providers from both the public and private sector who meet the accreditation requirements (Region Skåne, 2013; Vamstad & Stenius, 2015).

Patient choice involves a transfer of power from decision makers and care providers to patients. It is particularly important to study this transfer of power in the case of OST due to both the control and disciplining of patients that has often characterised this treatment method, and the fact that the treatment involves medicating with powerful pharmaceuticals that are also attractive on the illegal drugs market. A transfer of power from clinics to patients means that the latter choose their care provider when treatment is initiated, and that they can switch to a new care provider if they are dissatisfied. Care providers must therefore be attractive in order to be chosen by patients.

In an earlier study, Andersson and Johnson (2020) have analysed the significance and consequences of patient choice in OST from the patients’ perspective. The patients interviewed generally perceived an increase in empowerment and in their ability to influence their own treatment. Having the opportunity to choose and switch between treatment providers was appreciated by all the patients interviewed, including those who had not chosen to change clinics. The interviewed patients reported that they had experienced a greater influence over their treatment situation and that staff conduct towards them had improved by comparison with their previous experiences of treatment (Andersson & Johnson, 2020).

The aim of this article is to study the power shift produced by the patient choice reform from the perspective of the treatment providers. More specifically I will examine how the OST clinics view the empowerment experienced by patients, what this has meant in practice for their work, and what strategies they have employed to deal with the competition for patients. The study is based on interviews with clinic managers from all clinics that provided OST treatment in Skåne during the period 2014–2017. The interviews were conducted on two occasions, the first during the early phase of the implementation of the reform, and the second approximately two years later.

Examining the effects of patient choice for the OST clinics is of interest as a means of developing knowledge on the legitimacy of the reform from the treatment providers’ perspective. The question is also of particular interest given the reform's focus on increasing patient influence, which previous research on OST has raised as an important area for improvement (Deering et al., 2011; Frank & Bjerge, 2011; Kolind, 2007; McElrath, 2018). Treatment providers’ perceptions of the implementation of patient choice in OST are also of significance to the outcome of the reform in terms of the goals of increased empowerment, accessibility, and diversity. Furthermore, patient choice as a means of OST provision is an under-researched area, and improved knowledge is needed about the functioning of different forms of OST provision regarding both patients and treatment providers.

## Market orientation, empowerment, and patient choice in substance use treatment

The shift towards increased market orientation and privatisation of services within the welfare state was primarily driven by liberalisation and internationalisation in wealthy Western countries during the final decades of the 20th century (Blomqvist, 2004; Erlandsson et al., 2013; Fotaki, 2013). In Sweden, the move towards market orientation within the healthcare sector and other areas of welfare has been both swift and far-reaching by comparison with other European welfare states (Fredriksson et al., 2013; Sivesind & Trætteberg, 2017; Stenius & Storbjörk, 2020). Care provision for persons with substance use problems has also been affected by this trend, which has been studied by Storbjörk and colleagues (Storbjörk et al., 2019) in a research project on New Public Management (NPM) in municipal and regional substance use treatment. Among other things, these researchers have studied the strategies used by staff to deal with organisational tensions in NPM-like organisations (Storbjörk, 2020) and have compared the implementation of procurement regulations for substance use treatment in four Nordic countries (Stenius & Storbjörk, 2020).

Studies on market orientation in the field of substance use have been conducted in a number of countries, primarily in Europe and Australia. These studies tend to direct their principal focus at organisational policies linked to payment or reimbursement systems (Jones et al., 2018; Nesvaag & Lie, 2010; Roman et al., 2006; Shepherd et al., 2014; van de Ven et al., 2020), although some studies have focused on the effect of NPM on staff working in substance use treatment (Lewiskin, 2018). Few studies have focused on examining different forms of substance use care delivery from a staff or patient perspective however (van de Ven et al., 2020)*.* The delivery forms that often constitute the focus of studies in the field of market-based service provision are results-oriented models, such as payment by results, or public procurement, i.e., where there is competition to sign a contract regarding a certain segment of the care market (Goddard, 2015). In Sweden, studies of public choice of treatment provider, which is the form of care provision examined in this article, have primarily focused on primary care (Vengberg et al., 2019) and elderly care (Erlandsson et al., 2013), whereas none have examined substance use care (Storbjörk & Stenius, 2018).

One of the fundamental reasons for using patient choice for treatment provision is that it transfers power from politicians, officials, and staff to patients. Giving patients the ability to choose where they go for treatment produces the conditions for a more equal relationship between patients and care providers (Brekke et al., 2014; Vamstad & Stenius, 2015). In Sweden, patient choice is regulated by the Act on System of Choice in the Public Sector (2008:962). Based on the Swedish legislation, the patient choice system means that care may be provided by both public and private sector actors, inasmuch as they meet the accreditation criteria specified by the municipality or region managing the care. There is no restriction on the number of care providers that can establish themselves in a given area, and patient costs are the same irrespective of which care provider is chosen. In theory, patient choice systems assume that the market will then regulate itself, as low-quality treatment providers will not be chosen, and will in time therefore leave the market (Anell, 2013; Erlandsson et al., 2013).

In 2014, the then right-wing-led Skåne Regional Council, the third largest healthcare district in Sweden, decided to introduce a patient choice reform for OST (Region Skåne, 2013), which is described in more detail in an endnote.^2^ The aim of the reform was to increase the availability of OST, to improve patient empowerment and patients’ influence over their treatment, to improve staff–patient relations and to produce increased diversity among treatment providers. Since the introduction of the reform, OST providers in both the public and private sector must accept patients who choose their clinic without delay, provided the patient meets the criteria for OST determined by the National Board of Health and Welfare (NBHW; Socialstyrelsen in Swedish).^3^

Empowerment is a central concept in this study and is defined as involving individuals in a position of powerlessness or vulnerability gaining power that gives them the ability to emancipate themselves and obtain more control over their lives. Helping service users towards greater empowerment constitutes an important element in the work of care providers (Adams, 2008; Frank & Bjerge, 2011). From this perspective, the increase in patient empowerment produced by patient choice systems may be considerable since they give patients the opportunity to influence where they wish to be treated by choosing a care provider. For OST patients, influence and empowerment are particularly important because the treatment often constitutes an important part of their lives.

Price and quality constitute the two principal reasons for choosing, and leaving, a service provider (Hirschman, 1970; Vamstad & Stenius, 2015). Since payments for OST treatment provision following the patient choice reform are fixed based on Region Skåne's regulations, treatment providers do not compete on price but on perceived quality. Thus, the goal of clinics is to attract patients by designing their work and treatment provision in accordance with patient preferences, to avoid the risk of not being chosen. Further, patient choice systems are based on an idea that care providers will diversify, and that competition and choice will be based on different care providers varying their focus and services in order to attract different patient groups (Wisell et al., 2019). However, diversity may not necessarily be positive. One concern prior to the implementation of the patient choice reform was that clinics might compete on the basis of prescribing other prescription drugs in addition to OST pharmaceuticals (Region Skåne, 2013). This risk is discussed later in the article.

## Control and power imbalance in OST

There is a substantial body of research on the use of discipline and control in OST provision, and on stigmatisation and perceptions among patients of being subjected to ill-treatment and invasions of privacy (Harris & McElrath, 2012; Holt, 2007; Keane, 2009; Lalander, 2012; O’Byrne & Jeske Pearson, 2019; Petersson, 2013b). OST is characterised by greater inequalities in the staff–patient relationship than are found in most other care-provision relationships (Järvinen, 2013; Lalander, 2012; Lilly et al., 1999; Petersson, 2013b). Patients in OST are required to follow the rules of the clinics, which are often strict, and clinics have been able to punish patients in cases of non-compliance by means of supervising their consumption of medication or by imposing dosage restrictions (Bourgois, 2000; Dahl, 2019; Frank, 2020; Fraser & Valentine, 2008; Friedman & Alicea, 2001). OST has been described as the most highly regulated treatment method in modern medicine (Stoller & Bigelow, 2006) and has often been governed by detailed national regulations with regard to factors such as inclusion and exclusion criteria, requirements for patient attendance, dosage levels, medication collection routines, urine testing, record-keeping and treatment duration (Rosenbaum, 1995; Skretting & Rosenqvist, 2010).

Studies have shown that the use of control and highly regulated routines may reduce the willingness of opioid-dependent individuals to participate in OST (Peterson et al., 2010; Richert & Johnson, 2015). As a result of the strict regulatory framework surrounding OST, the work of OST professionals requires an ability to combine both control and supportive functions. A number of studies have examined OST clinics’ perceptions with regard to this duality (see, e.g., Bacon & Seddon, 2020; Lilly et al., 1999; Petersson, 2013a, 2013b). Lilly et al. (1999) have argued that there is a tension between the roles of being “gatekeepers” for methadone, and counsellors, since there is some level of conflict between the two. However, staff reported wanting to develop working methods that were able to integrate these two areas of responsibility. A study by Bacon and Seddon (2020) examined how control was exercised in substance use treatment, both in OST and in non-pharmaceutical treatment provision. For OST staff, there is an element of control in the treatment, and a clear power imbalance between staff and patients. Decisions relating to the provision of and restrictions regarding methadone emerged in the study as a particularly potent means of exercising power, e.g., withholding methadone in the case of lateness or intoxication (Bacon & Seddon, 2020). Petersson (2013b) also notes the unequal power relations between patients and treatment staff in a Swedish study of patient and staff experiences of everyday practice at OST clinics. The subordinate position of patients emerged particularly clearly in situations in which various forms of non-compliance were assessed by staff (Petersson, 2013b).

The controlling elements of OST are legitimised by reference to security concerns. Medication in the form of methadone or buprenorphine are provided on the basis of regulations that are intended to ensure that patients in need of OST receive safe treatment, but also that any diversion of substances to the illegal drug market is prevented. This makes the balance between the control and support functions of staff particularly challenging (Bacon & Seddon, 2020; Lilly et al., 1999). Another aspect of the controlling measures relates to the medical risks associated with the illicit use of certain other substances in combination with OST medications. The use of benzodiazepines during OST is common (Bramness & Kornør, 2007; Fatséas et al., 2009; Frank, 2020; Laqueille et al., 2008; Vold et al., 2020). Several studies have shown that simultaneous use of benzodiazepines and other depressant drugs increases the risk for overdose and opioid-related mortality (Brands et al., 2008; Jones et al., 2012; Macleod et al., 2019; Sun et al., 2017; Tjagvad et al., 2016).

OST has also been characterised by regulation and control in Sweden (Lalander, 2012; Petersson, 2013b). The treatment has been subject to strict regulations and restrictive programme-entry criteria, and patients have often had to queue prior to starting treatment. It has been possible to punish non-compliance, commonly in the form of the use of benzodiazepines and other drugs, by means of involuntary discharge followed by a suspension period (Johnson, 2007; Ledberg, 2017). The strict system of regulations, and the often long waiting lists, have intensified the unequal power relations between patients and staff, both in Sweden and in other countries (Harris & McElrath, 2012; Järvinen, 2013; Petersson, 2013b). A further aspect of the power imbalance involves supervised urine testing. Exercising control in the form of urine testing is a routine means of ensuring that OST patients are not using prohibited substances, and is often perceived as disrespectful and humiliating by patients (Friedman & Alicea, 2001; Lalander, 2016; Petersson, 2013b).

Over recent years there has been a shift in OST in Sweden from a strict approach in which using other substances was not accepted, towards lower thresholds for programme entry and higher thresholds for involuntary discharge, and towards a system in which harm reduction may be an explicit treatment goal. The focus on control has declined in favour of a focus on retention (Andersson & Johnson, 2018; Monwell, 2019). A revision of the national guidelines for OST that occurred in 2016 may be viewed as an adaptation to this shift (HSLF-FS 2016:1).^4^ The introduction of the patient choice reform in Skåne in 2014 may also be viewed as a step on the path from a stricter and more control-focused approach to OST towards a form of treatment provision characterised by greater accessibility, increased patient influence, and a greater tolerance of relapse and the (illicit) use of narcotics in addition to OST medications.

## Methods

### Data collection

The study data comprise interviews with clinic managers at the OST clinics in Skåne. The interviews were conducted on two occasions. The first interview was conducted shortly after the introduction of the reform on the basis of the manager’s expectations, hopes and concerns regarding the implementation of patient choice, the second approximately two years later, when the reform had become somewhat more established. The first wave of interviews was conducted during the period November 2014–April 2015, the second in December 2016–February 2017. The clinic managers were interviewed as representatives of the clinics at which they worked at the time of the interviews. Several of the mangers engaged in some level of clinical work in addition to their managerial responsibilities or had previously worked in OST as nurses or counsellors. All interview participants were front-line managers who worked on site at their clinics.

In order to obtain a holistic perspective on the managers’ views of the reform, all clinic managers were interviewed, with the exception of the manager of a private clinic that opened in the autumn of 2016 within a firm that already ran two clinics in the region.^5^ A total of 26 interviews were conducted; 12 in the first wave and 14 in the second. Some participants were responsible for more than one clinic, and the number of interviewees is thus smaller than the number of clinics included in the study. The first wave included 15 OST clinics (seven in the public sector, eight in the private sector), and the second 18 clinics (seven public sector, 11 private sector). All 13 first-wave interviews and eight of the second-wave interviews were conducted by the author. Björn Johnson conducted six of the second-wave interviews. In all but one case, the interviews were conducted at the participants’ workplaces. One of the second-wave interviews was conducted on Malmö University premises. The interviews lasted on average one hour and were recorded and transcribed verbatim.

The interviewers employed a semi-structured interview guide that had been formulated for the purpose of a large-scale stakeholder study of the patient choice reform (Andersson & Johnson, 2018). The interview guide covered the following themes: (a) treatment accessibility, (b) collaboration with other stakeholders, (c) competition and diversity, (d) psychosocial support and control, (e) patient empowerment and participation, (f) the freedom to choose and switch clinics, (g) medical security and benzodiazepines, (h) diversion and other treatment risks (i) the remuneration system. The second interview wave also included (j) changes in working methods and patient composition, and (k) the future of the patient choice system. For the purposes of this study, themes c, d, e, f, g and h have been of special interest.

### Analysis

The interviews have been analysed using a three-phase manual qualitative text analysis (see, for instance, Braun & Clarke, 2006; Moser & Korstjens, 2018). The first phase involved reading through the interview transcripts several times in order to identify themes based on the central content in the data, i.e. the content that appeared repeatedly during the interviews. This central content, and the themes included in it, was dominated by, but not limited to, the areas of the interview guide that constitute the study's focus. A second, more detailed coding was then conducted within the themes that had emerged in order to examine the various views and attitudes expressed by the interviewees in more detail. The themes into which the data were sorted largely correspond to the sub-headings employed in the results section. Finally, the transcripts were read once more in order to select representative quotations (Moser & Korstjens, 2018; Rennstam & Wästerfors, 2015). Most of the illustrative quotations presented below are drawn from the second wave of interviews. In cases where a quotation is from a first-wave interview, this is noted in parentheses after the quotation.

## Results

The results are presented under four sub-headings. I begin by focusing on patient empowerment, before moving on to opportunities to choose and switch clinics, and the issue of diversity. Then I take on the issue of control, and finally competition via the prescription of benzodiazepines. In those cases where there were clear differences between the first and second interview waves, or between clinic managers from the private and public sectors, I have directed a special focus at these.

### Patient empowerment and influence

One important incentive for introducing patient choice in OST was that patient influence within this treatment method has been highly restricted, at the same time as the treatment involves long-term and intensive contacts with treatment providers. In the interviews with clinic managers, there emerged a generally positive attitude towards patients being given increased influence over their treatment situation via the introduction of freedom of choice. During the first interview wave, this was expressed explicitly by several of the interviewees. Some linked their positive attitude to OST patients, and opioid-dependent individuals in general, being a neglected group within the care sector, who had for a long time had little influence regarding the treatments on offer. Others stated, on the basis of a more ideological perspective that viewed empowerment and freedom of choice as being positive per se, that the shift towards greater patient influence was something that should be happening both in OST and in other areas of care provision. The right of patients to participation and influence over their treatment is also prescribed by the legislation that forms the basis for healthcare in Sweden (The Patient Act (2014:821)). The positive attitude towards patient influence expressed in the interviews stands in contrast to what has previously characterised the general view of OST in Sweden, where the focus has instead often been directed at patient adherence to treatment rules, and the consequences of non-compliance (Petersson, 2013b). Some public sector clinic managers who had been providing OST for many years prior to the patient choice reform stated at the first interview that reviewing their work from a patient perspective and determining whether changes were needed was of value as a means of ensuring that their clinics would remain attractive.

Those of us in the public sector must stop and think about what we are good at and get our patients to feel happy about being where they are. […] What it is that we are doing well—it has been positive reflecting over how we work. (IP 1, public sector clinic, first interview wave)

One example that was raised by the interviewees related to opening hours for the collection of medication, which had generally become more generous since the introduction of the patient choice reform. The opening hours are about 8:00–16:00 on weekdays with extended opening hours one or two evenings a week. Before the reform, medicine should be collected before noon, which patients sometimes had difficulties conforming to. At weekends, most clinics are open in the mornings between 8:00–12:00 (where there are two or more clinics within the same owner provision there may be that only one of these has a reception open at the weekend).

The view that patient empowerment had increased since the reform also emerged clearly in the second-wave interviews. The general view among clinic managers was that this was a desirable change, and several linked this directly to the introduction of patient choice.

Lisa: This empowerment aspect, that there would be a bit more of a power shift from the clinic to the patients [in connection with the introduction of patient choice]?

Interviewee: But I think that I’ve, it's really changed, and that's positive. Very positive.

Lisa: In what ways do you see that?

Interviewee: You can see it in the way that they [the patients] are like more, they make the demands they have a right to, which they maybe didn't do before in the same way. They maybe didn't even know what demands they could make. (IP 9, private clinic)

Some clinic managers mentioned that the increase in patient influence over treatment, and the opportunity to make “demands”, meant that patients also assumed more responsibility and started feeling more like patients than “drug addicts”. Other interview participants placed a special emphasis on the freedom of choice that had been introduced with the reform. These managers viewed the patients’ ability to choose which clinic would provide treatment, and to move to a different clinic, as being important psychologically, which is very much in line with the views that patients expressed in a previous study (Andersson & Johnson, 2020).

A few interviewees from public sector clinics expressed a positive view of the shift towards increased patient influence, but at the same time said that this shift had already started prior to the patient choice reform. One clinic manager with substantial experience argued that there had been successive changes in the field of OST and expressed doubts as to the significance of patient choice for these developments. Others said that they had seen signs of a tendency towards increased patient influence prior to patient choice but that the reform had accelerated this trend.

The interviewees consistently described an awareness of the importance of treating patients with respect and of being judged as treatment providers on the basis of this parameter. In this way, competition becomes an incentive for the clinics to look after their patients and to design their work in accordance with the increased focus on patient influence that is implicit in the freedom of choice associated with the reform.

### Choosing, switching and diversity among OST clinics

The opportunity for patients to choose their treatment provider and to switch to another if they are dissatisfied constitutes the central mechanism in a patient choice system and provides the basis for competition among treatment providers. There is also an aspiration to produce some form of diversity among treatment providers within the system, i.e., that these differ from one another in a way that allows patients to choose between clinics on the basis of different approaches to treatment or the provision of a varied range of services.

The participants’ views on diversity regarding both treatment content and the clinics’ target groups were discussed during the first wave of interviews. Two of the clinic managers described that their newly established clinics were planning to have a special focus on women, while another spoke of a niche directed at patients with ADHD problems. One manager said that the clinic had already had a special focus on patients recruited from needle exchange programmes prior to the reform. A couple of clinics were planning to focus on opioid-dependent persons with pain problems. The manager of one clinic said that they had a special focus on the patients’ health in a broader sense, and that they provided many activities in addition to their medical provision. Another clinic had started enrolling patients with high treatment compliance in a separate unit. Several clinic managers described measures involving support groups for patients on different themes, in part based on patient requests. One difficulty, however, was getting patients to want to participate in group activities.

It's not so easy to diversify. If you start groups, then a few come to begin with, then three turn up, then one or two and so you have to close the group down. (IP 6, public sector clinic, first interview wave)

In the second-wave interviews, it became clear that in addition to differences in views on the prescription of benzodiazepines, which are discussed below, little had happened in terms of diversification. Most of the clinic managers felt that diversity was something positive in principle, and that it was regrettable that it had not occurred in line with the intention of the reform, as is exemplified in the following quotation.

Then I think it would be really good if we could diversify among ourselves. If someone is really good for ADHD patients, and someone is really good for women … That was what I thought when I heard about patient choice. It's a really smart idea—we were a huge clinic and that's not good. (IP 8, public sector clinic)

One type of diversity that emerged from the interviews, and a factor that affected patients’ choice of clinic, was the clinics’ approach to the possibility of prescribing benzodiazepines, which is discussed in more detail below. Several clinics were concerned during the initial phase following the patient choice reform that having a restrictive approach to prescribing benzodiazepines would affect the possibility of attracting a sufficient number of patients. During the second-wave interviews, several clinic managers mentioned that they had previously been worried about whether their clinics would survive if patients were to use their new freedom of choice to switch to a clinic with a less restrictive approach. These concerns then successively diminished when it became apparent that many of the patients were not choosing to leave their current clinic, and that there were enough patients for all clinics. In a recent Swedish study on patient choice in primary care, care providers reported that their patient lists were stable and that they were not experiencing competition for patient numbers to any major extent (Vengberg et al., 2019). If someone left to join another care provider, a new patient soon came to fill the vacant place.

Following the implementation of the patient choice reform, the public sector clinics lost some of their patients, but there was an increase in total patient numbers in Skåne that compensated for this. At the second-wave interviews, several clinic managers stated, sometimes with surprise, that patients were choosing to apply to their clinics, and to remain with them, despite their having a restrictive approach to prescribing benzodiazepines and other substances.

We are incredibly restrictive with benzos here and they know that when they apply to come here too. The first thing we do is to tell them this, and they often know about it, have heard about it. […] And a lot [of people] apply to come here for that reason, because then you think that you will be able to get a “cleaner” form of treatment on that basis as well. (IP 11, private clinic)

This was something that also emerged in interviews with patients in a previous study on the patient choice reform (Andersson & Johnson, 2020). Even though the patients in that study were positive about having the opportunity to be given access to benzodiazepines when needed, the majority wanted to be treated at a clinic with a more restrictive approach to these substances.

The majority of moves between treatment providers occurred during the first two years of the reform. Since then, patient numbers at the different clinics have remained relatively stable and switches between clinics have become less common. According to annual surveys at the clinics, 131 patients had used their opportunity to change clinics in 2015 (out of a total of 1,237 patients in OST in the region that year), of whom 106 switched from publicly run clinics. Fewer changed clinics the following year, 88 patients, despite a higher total number of patients (1,453). Of those 88, 48 switched from public clinics. Complete figures for 2017 are missing, but reported data indicate that the trend of fewer moves persists (Andersson & Johnson, 2018). At the time of the second interview wave, the number of patients switching clinics had thus declined, but patients sometimes threatened to move to another clinic in connection with a more transient dissatisfaction regarding some aspect of the treatment contact.

Lisa: Do patients threaten, “If I don't get this, then I’m going to switch clinics”?

Interviewee: Yes, but in that case, they can move to another clinic. […] It can happen [that patients threaten to switch clinics], and sometimes in the heat of the moment they can sometimes say “in that case I’m switching clinics”, “Yes, and you’re very welcome to do so; you know where the forms are; all you have to do is apply”. But then they often come back the next day and say, “No, I was only kidding, I was just angry”. (IP 3, public sector clinic)

That clinic managers take such threats as lightly as is shown in the above quotation testifies to a feeling of confidence that most patients are satisfied with their treatment situation and will stay, even though a patient might occasionally go through with a threat to leave the clinic. Ideally, an exchange such as that described in the quotation would lead to a conversation between the patient and the treatment provider about the cause of the patient's dissatisfaction and how the clinic might respond to this. As has been noted in a previous study on patient perceptions of the patient choice reform, the ability of patients to use their “voice” and to protest at perceived injustices has increased (Andersson & Johnson, 2020), which represents a change by comparison with findings from research on OST prior to the reform, when patients who were classified as “difficult” might risk being discharged (Petersson, 2013a).

### Control and medical security

In OST, control in the form of medical security primarily relates to the use of drug analyses to follow up on medication and to verify that patients are abstinent from other drugs, and supervising the taking of medications, or the daily collection of medication, to reduce the risk of pharmaceuticals being diverted. Thus, security relates both to OST patients and persons outside treatment who want to obtain these medications on the illegal market. Due to the inherent risk that OST may entail for opioid-dependent people both in and out of treatment, OST patients are considered less able to manage their medication themselves in comparison with other patient groups, which is contrary to the foundations of the idea of increased patient influence and self-management. When the strong focus on control that OST includes needs to coexist with ideas about empowerment and emphasis on patient influence, it therefore entails special dilemmas for staff in the everyday institutional practice at the clinics (Frank & Bjerge, 2011). The first-wave interviews took up the balance between control and psychosocial support in OST treatment. The general view was that some level of control was necessary since the treatment involves potent narcotic substances. At the first interview, some clinic managers, primarily from newly opened private sector clinics, said that there were already signs of a positive shift from a focus on the collection of medication and controls in the form of urine testing to an increased focus on psychosocial and supportive aspects, which might be interpreted as a shift in the role of OST staff from being “gatekeepers” towards a more counsellor-focused role (Bacon & Seddon, 2020; Lilly et al., 1999).

A somewhat different view and approach to control emerged during the second-wave interviews. Following the NBHW's revision of the national OST guidelines in 2016, repeated positive drug tests are no longer a cause for involuntary discharge. This change occurred once the patient choice reform had been in place for a couple of years, but the successive trend towards a less restrictive view had started even prior to the implementation of the reform. Patients may now remain in treatment if they test positive for drugs, but are required to collect their methadone or buprenorphine from the clinic on a daily basis. The requirement for daily visits to collect medications following positive drug tests was applied by all clinics. Older patients with a “chronic” use of benzodiazepines or other substances for many years are also covered by the requirement to collect OST medications daily. One manager who worked at a “restrictive” clinic said that the clinic's staff ignore the illicit use of benzodiazepines by some patients with a long history of substance use and a vulnerable life situation.

Interviewee: I mean we have a few where we, yes, they’re not going to stop taking benzos. We turn a blind eye to the fact that they are getting hold of these themselves.

Lisa: They come, and they give positive urine tests, but they remain in treatment?

Interviewee: Yes, and then come every day. (IP 2, public sector clinic)

This type of use could previously result in involuntary discharge. These patients are now able to remain in treatment, but at the price of having to make daily visits to the clinic to collect their medication. Previous research has shown that a barrier for opioid-dependent people to seek treatment has been that they feel dependent on the clinic instead of an illegal drug (Harris & McElrath, 2012; Peterson et al., 2010; Richert & Johnson, 2015), partly because they must attend the clinic at specific times in order to collect their medication. That patients now remain in OST to a greater extent is positive for the patients in relation to the alternative, which would mean discharge. At the same time, the condition of having to collect their medication every day may be seen as being in conflict with the patient choice reform's aim of empowering patients and increasing their influence over their treatment. An economic aspect of patient visits may also be mentioned since the clinics receive compensation for each individual patient visit.^6^

A change in the way drug tests were conducted also emerged between the first and second interview waves. During the first-wave interviews, only one clinic used saliva tests as an alternative to urine testing. By the second wave, this practice had spread to several clinics, partly as a means of using less invasive control methods.

We mostly have urine tests, but we have started taking [saliva tests], because some patients find it very difficult to pee and it is distressing for them to stand here, and there's a lot of people around and they're pulling on doors and so on. (IP 9, private clinic)

The forms used for drug testing were one area in which OST patients expressed a desire for change in the context of an evaluation of the patient choice reform (Andersson & Johnson, 2018). A move towards providing alternative types of drug testing to urine tests may be viewed as a means of striking a balance between maintaining continued high-level medical security while improving patient influence over their treatment situation. The diversion of methadone and buprenorphine to the illegal drug market, and the associated possibility of overdose mortality, was viewed as a risk prior to the introduction of the patient choice reform. An awareness of this risk emerged in both interview waves. The general view among those interviewed during the second wave was that the risk for methadone and buprenorphine diversion had not increased since the reform, despite a larger number of patients being prescribed these medications and an increased tolerance towards the use of other narcotics during OST. Some clinic managers raised the possibility of prescribing buprenorphine-naloxone in sublingual film as a means of reducing the risk for diversion and overdose. The fact that many patients make daily visits to the clinics as a result of the use of other prohibited substances may also play a role in the extent to which OST medications reach the illegal drug market.

### Competition through benzodiazepines

The issue of benzodiazepines has already been touched upon. Benzodiazepines constituted a very significant theme in the interviews, however, and were raised spontaneously by many of the clinic managers. During the first-wave interviews, concerns were raised that medical decisions might become a means of unfair competition, primarily by managers at public sector clinics. Even at their second interviews, some of the participants raised concerns that patients would choose to switch clinics if they did not receive the medications they desired, as exemplified by the following quotation.

It's quite right, I think, that patients should have influence—that's something you should have irrespective of where you are in the care system. With patient choice it would be easy for, how should I put it, for the patients to use this right to: “Yeah but I’m going to have that medication or I’m not going to come to you”. And then things have gone awry. They don't have the knowledge and the competence to make that assessment sometimes. […] We’ve lost some [patients] because of it [the possibility of switching clinics]. Both lost, and also patients that come here and like already have a list of demands. […] It's a bit difficult to deal with actually. (IP 8, public sector clinic)

There was a consensus among the “more restrictive” clinics (both public and private sector) that the patients who left their clinics primarily did so in order to have access to other medications than those they had been given at the clinic.

Björn: If patients move from your clinic, what reasons do they give? Or what is it about? Is it conflicts, or …?

Interviewee: It's benzodiazepines, in any case for us. Not conflicts. (IP 7, private clinic)

A previous study of patient perceptions also found that benzodiazepines were a reason for choosing and switching clinics (Andersson & Johnson, 2020). Two private sector clinics differed from the majority regarding their more permissive view on prescribing benzodiazepines. These clinics have been subject to both regional and national investigations, and have been required to adjust their working methods, which involved the long-term prescription of benzodiazepines. Several of the other clinics, particularly in the first-wave interviews, directed strong criticism at the fact that there were clinics that in their view were competing with benzodiazepines and medical decisions. Even at most of the “restrictive” clinics however, there was some limited prescribing of benzodiazepines and Z-drugs, motivated as being necessary in certain cases of poor mental health or for older patients who had been using such substances for decades. In those cases, it was sometimes regarded as legitimate to deviate, in a controlled way, from an otherwise strict approach, even at clinics that regarded themselves a “benzo-restrictive”.

I mean, benzos … We know that there are clinics that are zealously zero. And there are those that prescribe in order to attract patients. If we think that there is a patient who really, really has to have it, you know, has chronic panic disorder, or almost an anxiety psychosis and, or has taken them since they were five years old, always. […] There aren't many, but there are a few [who are given this type of prescription at the clinic in question]. (IP 6, public sector clinic)

As has been mentioned, alongside freedom of choice, competition is the driving element in patient choice as a care delivery system. A certain change could be seen between the first- and second-wave interviews in the managers’ views of other clinics in terms of competition. During the second wave more managers emphasised that they did not view one another as competitors, but more as collaborative partners who were working to achieve the same goal. Despite this change in view, which was probably at least in part due to the fact that all of the clinics had been able to continue their work with a sufficient number of patients, a number of managers continued to raise the criticism that other clinics were in their view engaging in unfair competition on the basis of benzodiazepines.^7^ In general, however, the clinic managers felt that a more uniform approach to the prescription of benzodiazepines had developed, which they said the patients were also aware of.

## Discussion

Achieving a shift in the balance of power by improving the empowerment and influence of patients may be difficult in a treatment, such as OST, that is associated with strict regulations and control. Frank and Bjerge (2011) have examined the dilemma between, on the one hand, a focus on control in treatment and, on the other, empowerment and greater equality in the relationship between staff and patients. They note that reforms are introduced in settings that are affected by current and former policies and traditions, which must be taken into consideration when examining the outcome of a new policy or reform. The OST patient choice reform in Skåne was implemented at a time when a shift towards a less strict approach to regulations and practice was already underway in OST, and in a region that had a relatively liberal and user-oriented view of drug policy by comparison with Sweden as a whole. At the time of the reform, however, OST was still mainly conducted on the basis of a high-threshold perspective, and the availability of treatment places was relatively limited.

Increasing the empowerment and influence of patients and other service users in the welfare sector may be achieved in different ways. In the reform examined here, the mechanism intended to produce the shift towards greater patient empowerment has involved giving patients the opportunity to choose and switch between treatment providers (Brekke et al., 2014; Vamstad & Stenius, 2015). The interviews with clinic managers indicate a generally positive attitude towards increased patient empowerment in OST, and also that there has been a shift in this direction following the introduction of the patient choice reform.

The fact that patients are now able to choose their clinic has thus given them greater influence over their treatment. At the same time, the clinics’ ability to plan their work on the basis of patient numbers has been reduced, since the patient choice system is based on patients being able to choose the treatment provider they find most attractive. The clinic managers expressed an acceptance of this change, which for them means being exposed to competition and with this the risk of not being chosen (Anell, 2013; Erlandsson et al., 2013). This attitude among the managers was in part based on a perception that it is reasonable for patients to have more influence over their treatment situation, but also a perception that competition may in part be viewed as positive, and as serving as an encouragement to review their operations and working methods.

One more tangible change that has occurred since the introduction of the reform is that some clinics have started conducting drug testing on the basis of saliva rather than urine testing. Increased patient influence may have played a role in this, with clinics introducing strategies to adopt less invasive forms of control as a means of improving their competitiveness (Andersson & Johnson, 2020; Monwell et al., 2018). As has been noted, control constitutes an important element in OST, and a system of control measures must be integrated into the system for reasons of security. A well-functioning balance between caring and control-focused activities is also important for OST staff (Bacon & Seddon, 2020; Lilly et al., 1999). Prior to the implementation of the reform, concerns were expressed that competition would lead to a decline in medical security in OST treatment (Region Skåne, 2013). Based on the interviews with clinic mangers, this risk does not appear to have been realised. Instead, the interview data show that levels of medical security have remained high, and clinics have a similar approach to the use of prohibited substances, for example.

Concerns were also raised prior to the introduction of the reform that clinics would compete for patients by liberally prescribing benzodiazepines. This was viewed as one of the greatest risks associated with the reform (Region Skåne, 2013). The issue of prescribing benzodiazepines during OST was also one where several clinic managers felt that patient influence might have become too great. As was noted in the introduction, the use of benzodiazepines during OST has been associated with poorer treatment compliance and a risk for overdose and mortality (Brands et al., 2008; Jones et al., 2012; Laqueille et al., 2008; Macleod et al., 2019). The prescription of benzodiazepines and similar substances by Swedish OST doctors has been restrictive (Andersson & Johnson, 2018; Nilsson, 2017), and as a result the use of benzodiazepines during OST has primarily occurred via illicit means. At the same time, OST patients have called for benzodiazepines to be prescribed in order to avoid the risk for involuntary discharge if a drug test were to find traces of these substances (Petersson, 2013a, 2013b).

The fact that certain service providers offer attractive goods or services in addition to those they have undertaken to supply as accredited service providers may be viewed as a form of negative or unfair competition (Vamstad & Stenius, 2015). For some clinic managers, concerns regarding unfair competition in the form of prescribing benzodiazepines, as perceived by the majority of public sector and also some private sector clinics, was linked to an anxiety that their clinics might not survive. Some of the public sector clinics that had existed for many years prior to the reform lost a large proportion of their patients when patient choice was first introduced, so their concerns were not entirely groundless (Andersson & Johnson, 2018). Follow-ups of patient numbers, and the second-wave interviews, showed however that there were sufficient patient numbers for all clinics to continue their activities, and these anxieties gradually decreased.

In the later interviews, several participants touched upon the change in the NBHW's guidelines for OST, which were then imminent and meant that concomitant use of prohibited substances would no longer be a cause for involuntary discharge. While this change was viewed positively, the clinic managers also saw problems with a situation where an increasing number of patients would use illicit substances for a length of time. Several clinics were discussing how treatment could be provided in a medically secure way, and how they might work to motivate this group of patients to reduce or to desist from their long-term use of benzodiazepines. There is no international consensus on how the use (illicit or prescribed) of benzodiazepines during OST should be handled. A small number of studies have examined maintenance treatment with benzodiazepines in the context of OST, but without producing unequivocal results (Bakker & Streel, 2017; Weizman et al., 2003). A study by Macleod et al. (2019) found that the prescription of benzodiazepines in OST was associated with higher treatment retention, but also with a higher risk for overdose mortality. Vold et al. (2020) note that the prescription of benzodiazepines among OST patients in Norway is very common. Both these authors, and other researchers, have called for more well-designed studies on the co-prescription of benzodiazepines during OST.

One reason for the clinic managers’ positive attitudes towards the patient choice reform was the opportunity for diversity in the range of treatment services clinics were expected to provide. Apart from the liberal approach to prescribing benzodiazepines found at a couple of clinics, however, there appeared to be no obvious diversification among the treatment providers. During the second-wave interviews it became clear that the attempts that had been made towards diversifying had not been integrated into the clinics’ activities, despite the interviewees’ positive attitudes towards diversity. However, it is difficult to acquire competition-based diversity in a treatment method such as OST in the way freedom-of-choice systems are in theory intended to. OST is provided based on a strict regulatory system that restricts how clinics can choose to conduct treatment activities. Further, the remuneration system is designed to facilitate the clinics’ work with resource-intensive patients by ensuring that this patient group generates higher levels of remuneration than patients with high compliance and thus a low frequency of treatment contact. This reduces the incentive to diversify treatment provision based on a focus on different patient groups. Wisell et al. (2019) note that diversity has been difficult to achieve in other welfare areas in Sweden such as primary care and community pharmacies as well. They argue further that the definition of diversity employed by policy makers is vague. Their study also found that there was a lack of clarity from policy makers regarding whether diversity should be viewed as an effect of, or as a means to reach, competition, which led to ambiguities regarding the way in which competition might result in diversity (Wisell et al., 2019).

In an earlier study, Andersson and Johnson (2020) examined patients’ perceptions of the patient choice reform in OST. Viewed in relation to the current article, the areas of interest were mainly the same for both stakeholder groups, and they also had similar views in these areas. Both clinic managers and patients felt that increased patient empowerment was positive, even though the two groups differed somewhat in their views on how far patient influence should extend. Both groups emphasised the psychological importance for patients of the knowledge that they can switch between clinics should they want to. A consensus can also be noted in relation to benzodiazepines, in the sense that both patients and clinic managers expressed a negative view of excessively liberal prescription practices.

A study by Deering et al. (2011) in which treatment providers, OST patients and opioid users were interviewed about measures to reduce barriers to OST, also found a consensus among these stakeholder groups in several respects. All three called for more flexibility and a less stringent regulation system, while the patients also wanted to be treated with greater respect.

Since OST reduces the risk for opioid-related mortality, it is important that it is delivered in a way that attracts patients, both to enter and stay in treatment (Cousins et al., 2016; O’Byrne & Jeske Pearson, 2019; Sordo et al., 2017). Previous research showing that individuals with opioid dependency have chosen to stay away from OST as a result of the strict regulations and a lack of respect from OST staff (Peterson et al., 2010; Richert & Johnson, 2015) illustrates the need to make OST more attractive. The aim of introducing patient choice into OST was to make this treatment method both more accessible and more attractive in the region by increasing both the number of available treatment places and also patient influence in the form of a transition of power towards the patients. This article shows that treatment providers have accepted and generally have a positive attitude regarding this shift towards more patient influence and the effect it has had on their own treatment provision.

## Conclusion

The clinic managers interviewed in the study have a generally positive attitude towards greater empowerment and the shift in the power balance towards patients that the patient choice reform has resulted in. The competition that has emerged in connection with the reform has primarily taken the form of different views on and approaches to prescribing benzodiazepines. A liberal approach to prescribing benzodiazepines was regarded as unfair competition by several clinics with a more restrictive prescription practice, and initially caused concern and dissatisfaction among these clinics. The reform's goal of increased diversity in treatment content has not been realised. With the exception of a variation in the approach to benzodiazepines, there has been no diversification. This is perhaps not surprising, however, given that OST is provided on the basis of strict national regulations, and that the design of the remuneration system provides no incentive for diversity.

### Strengths and limitations

One limitation that should be noted is that changes in patient influence and in views on the control exercised by OST staff may also be due to factors other than the patient choice reform. The revision of the national guidelines for OST in 2016, and the fact that there has been a trend towards a more harm-reduction-oriented view of this treatment method in Sweden, make it difficult to distinguish the effects of the patient choice reform from those of other, parallel changes in OST practice and policy.

One of the study's strengths is that all clinic managers in the region were interviewed twice. Only a relatively short time had passed between the introduction of the reform and the second interview wave, however. The study only included clinic managers. Interviews with other OST professionals may have provided a complementary view. This possibility was considered but not chosen for reasons of time, and because an important part of the study involved developing a picture of the consequences of the system change for the individual clinics, an issue into which the clinic managers may be assumed to have the best insight.

It is important to emphasise the study's limitations with regard to generalising the findings. It is not clear that patient choice would function in the same way in another area of the welfare system. It would be of interest to follow up attitudes towards the reform once OST has been delivered on the basis of patient choice for a longer time.

## Notes

1. In Sweden, healthcare is decentralised and managed by 21 self-governing regional councils with financial and organisational responsibility for healthcare in the respective region. Healthcare can thus be conducted in different ways with regard to region and type of care. Laws, central national governing documents, and guidelines regulate the regions’ obligations and, to varying degrees, the provision of care. Regarding OST, the National Board of Health and Welfare's guidelines are the national governing regulations that regulate conditions for inclusion and exclusion from treatment as well as the essential treatment design. Healthcare services are mainly provided by public care providers, but regions can out-contract care to private care providers in accordance with public choice of treatment provider or public procurement legislations. Patient fees are the same regardless of type of provider.

2. Patient choice within OST is regulated by accreditation requirements which, among other things, specify the level of staffing and competence required by an OST clinic and the services that a clinic must offer. Patients who meet the inclusion criteria may not be denied treatment. Patients may change to a different clinic at most once every six months. The aim of the provision specifying this time restriction, which may be viewed as a limit on the freedom of choice, is to provide the clinics with a real opportunity to develop a treatment alliance, and to prevent hasty and ill-considered moves between clinics.

3. According to the current guidelines for OST published by the National Board of Health and Welfare (HSLF-FS 2016:1), patients must be at least 20 years old and must be assessed to have been opioid-dependent for at least one year in order to enter treatment.

4. The most important changes introduced with the revision of the national guidelines for OST in 2016 relate to inclusion and exclusion criteria. A restriction regarding the type of opioid dependence that was required to be eligible for treatment was removed (the previous guidelines related only to “opiates”), and the criterion for entering treatment was changed from having a documented opioid dependency of at least a year to having been assessed by a doctor to have been opioid dependent for this period of time. The three-month suspension period following (involuntary) discharge was abolished, and relapse into the use of prohibited substances is no longer associated with being discharged from treatment in the way that was previously the case.

5. At one clinic, the manager was not available for interview during the second wave, and another employee with a long history of working at the clinic was interviewed instead.

6. The patient choice reform is based on a visit- and performance-based system of remuneration, in which different types of registered visits generate a pre-specified remuneration. The clinics are not guaranteed any patients, with remuneration instead being based on the individual patient choosing the clinic in question. The treatment is financed from regional taxation and is in principle free of charge to the individual patient. The remuneration provided by Skåne Regional Council is the same for all treatment providers and is paid retrospectively on a fee-for-service basis. One advantage with the fee-for-service system is that it reduces the incentive to avoid vulnerable or resource-intensive patients, which was one of the factors included in Region Skåne's considerations when formulating the remuneration system (KEFU, 2015).

7. In 2018, Region Skåne established regional guidelines for benzodiazepines in OST. These guidelines state that OST patients should not be treated with benzodiazepines, pregabalin or Z-drugs in outpatient care, and that in those cases where tapering is required, this should take place in an environment in which the patient can be continuously monitored, such as inpatient care or a treatment institution.
